# The Obstetrical Society

**Published:** 1898-10-22

**Authors:** 


### THE OBSTETRICAL SOCIETY.

At the last meeting of the Obstetrical Society the treatment of puerperal septicæmia by anti-streptococcus serum was brought up by Dr. J. Walters and Mr. A. R. Walters, who related a case dealt with in this manner, in which they considered there could be no reasonable doubt that the recovery was entirely attributable to the use of the serum. In the discussion which followed, the feeling was by no means unanimously in favour of

* *Lancet*, October 15th.

the method, and among other things it was urged not only that it was important to know what microbe was doing the mischief before using the anti-toxin, but that in many cases more than one microbe was at work. The remarks of the President (Dr. Cullingworth) probably best expressed the position which this treatment should occupy. Going on the general ground that streptococci were usually present, he could not agree with the opinion that the serum should not be administered until it had been definitely ascertained what was the offending microbe, and he thought the serum should be given without waiting for bacteriological investigation. At the same time the administration of the serum must never be allowed to be an excuse for leaving other things undone. The safe rule was to give an anæsthetic, to explore the uterus digitally, to remove all decomposing *débris* with the finger, clearing it carefully away, and not placing reliance on the douche alone, and, in addition to all this, to administer the serum without delay.

